# How Physiology and Pharmacology Shape Liver Regeneration: From Molecules and Cells to Trials

**DOI:** 10.3390/ijms27135967

**Published:** 2026-07-03

**Authors:** Amedeo Lonardo, Ralf Weiskirchen

**Affiliations:** 1 Independent Researcher, 41100 Modena, Italy; 2 Institute of Molecular Pathobiochemistry, Experimental Gene Therapy and Clinical Chemistry (IFMPEGKC), RWTH University Hospital Aachen, D-52074 Aachen, Germany; rweiskirchen@ukaachen.de

**Keywords:** clinical trials, liver regeneration, molecular physiology, pharmacology

## Abstract

Liver regeneration is a tightly orchestrated compensatory response in which the remaining liver regains mass and function after liver injury or surgical resection. However, it does not fully restore its original anatomy. This process involves sequential priming, proliferative, and termination phases that are controlled by inflammatory cytokines, growth factor signaling, metabolic regulation, and extracellular matrix remodeling. Clinically, regenerative capacity plays a crucial role in situations such as partial hepatectomy, acute liver failure, and chronic liver disease. Unfortunately, it can be compromised by conditions like cirrhosis, steatosis, diabetes, and other systemic or local disorders. Current strategies to improve regeneration, such as portal vein modulation, cell-based therapies, organoid technology, and bioartificial liver support, show promise. However, they are limited by challenges including impaired engraftment, functional immaturity, technical complexity, and scalability restrictions. Against this background, the present review explores the pathophysiological basis of liver regeneration and evaluates the clinical evidence on nutritional and pharmacological interventions aimed at enhancing the natural liver repair mechanisms.

## 1. Introduction

Liver regeneration is a highly coordinated physiological process in which the residual hepatocyte population proliferates and undergoes structural remodeling aimed at restoring hepatic mass and functional capacity after liver injury [1]. Liver regeneration is more accurately described as compensatory hyperplasia that, in contrast to true anatomical regeneration, does not restore the liver to its original gross anatomical configuration [2]. The liver’s extraordinary regenerative capacity had already been captured in antiquity through Greek mythology describing the myths of Tityus and Prometheus whose livers grew back every night [3].

In clinical practice, liver regeneration occurs following hepatic injury caused by a variety of noxious stimuli in those situations requiring the replacement of lost or damaged hepatic tissue. These include partial hepatectomy (due to malignant disease or during living-donor liver transplantation), acute liver failure, drug-induced liver failure, or chronic liver disease and cirrhosis [4,5]. In turn, dysregulated liver regeneration is a cause of severe chronic liver disease.

Current strategies for promoting liver regeneration are limited by significant biological and practical constraints. While the liver can efficiently recover after acute injury, chronic conditions like cirrhosis create a hostile microenvironment that hinders hepatocyte engraftment, proliferation, and restoration of metabolic function.

Non-autologous cell-based therapies face challenges such as substantial cell loss during cryopreservation, low engraftment efficiency, and the requirement for long-term immunosuppression [6]. Additionally, interventional radiologic and surgical approaches like portal vein embolization (PVE) and associating liver partition and portal vein ligation for staged hepatectomy (ALPPS) aim to stimulate liver regeneration by promoting hypertrophy of the future liver remnant through redirecting portal blood flow [7]. However, these procedures may be less effective in patients with pre-existing systemic or local conditions like diabetes, severe steatosis, or cirrhosis, which reduce regenerative capacity [8,9]. The rapid hypertrophy induced by these techniques may also lead to the development of functionally immature hepatocytes, delaying the recovery of essential synthetic and detoxification functions [10].

To address the limitations of single-cell transplantation, current research is focusing on three-dimensional liver organoids and bioartificial liver devices. However, these technologies face challenges in replicating the complex vascular and metabolic architecture of the adult human liver, as well as in large-scale manufacturing and standardization, which are significant barriers to clinical translation [11].

Within this complex landscape, this review aims to specifically explore the pathophysiology of liver regeneration, with a particular focus on clinical studies examining nutrient supplementation and pharmacological interventions aimed at enhancing the effectiveness of spontaneous hepatic regenerative responses. Given that cell-based therapies were addressed in a recent review [12], their consideration is beyond the scope.

## 2. Methods of Bibliographic Research

The identification of the relevant literature for this review was conducted through a structured bibliographic search primarily using the PubMed/MEDLINE database.

Clinical studies on liver regeneration were identified using “liver regeneration” in title and title/abstract searches, with clinical trial and 10-year filters applied where appropriate. Titles, abstracts, and relevant full texts were screened for human studies addressing surgical, pharmacologic, nutritional, metabolic, regenerative medicine, or biomarker-related factors influencing liver regeneration.

Additional publications were identified through manual cross-referencing of bibliographies in key articles and review papers. This snowball approach allowed the identification of earlier landmark studies and important clinical observations that might not have been captured by the primary search filters.

The literature search was last updated in May 2026. The initial PubMed/MEDLINE search using the term “liver regeneration” in the title field identified approximately 220 publications. After applying filters for clinical trials and screening titles and abstracts for relevance to human liver regeneration, about 60 potentially relevant studies were retained for further evaluation. Full-text assessment resulted in the selection of a smaller group of studies that were considered most informative for the present review. Additional relevant publications were identified through manual cross-referencing of reference lists in key articles and reviews. Because this work was conceived as a narrative review rather than a systematic review, the selection process was guided by clinical relevance, methodological quality, and the ability of individual studies to illustrate important mechanistic or translational aspects of liver regeneration. Studies focusing exclusively on experimental animal models without translational relevance, purely mechanistic in vitro investigations, or reports lacking sufficient methodological detail were generally excluded from the clinical evidence synthesis, although selected experimental studies were occasionally cited to provide mechanistic context where appropriate.

The final body of literature included in this review therefore reflects a combination of systematically retrieved clinical studies and additional relevant publications identified through reference tracking and Reviewer comments during peer review. This approach was intended to provide a balanced overview of both established and emerging clinical evidence regarding physiology, pharmacology, and therapeutic modulation of liver regeneration.

## 3. Molecular and Cellular Physiology of Liver Regeneration

Liver regeneration is a highly coordinated biological response that enables the restoration of hepatic mass and function following tissue loss or injury [13]. Instead of representing true anatomical regeneration, this process is best described as compensatory hyperplasia in which the residual hepatocyte population proliferates and reorganizes to re-establish functional capacity without fully recreating the original hepatic architecture [13]. Clinically, the efficiency of this regenerative response is a critical determinant of outcome after major hepatectomy, living donor liver procurement, and acute or chronic liver injury [13]. A wide spectrum of molecular pathways, cellular interactions, and systemic factors contribute to the regulation of this complex process.

**Figure 1:**
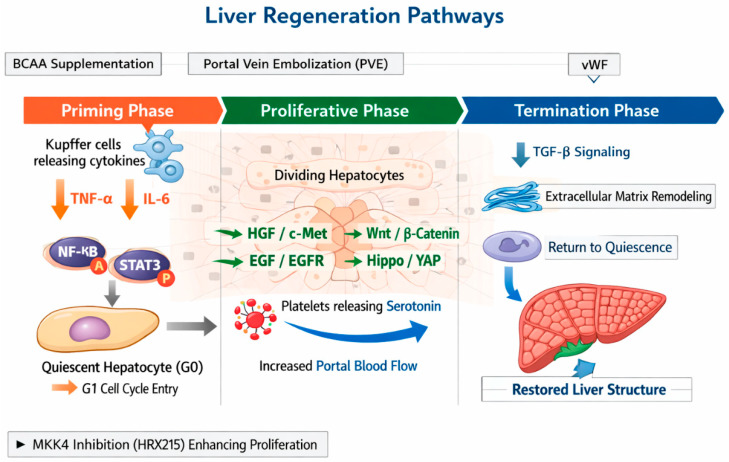
**Major molecular pathways regulating liver regeneration**. Liver regeneration is a coordinated process that restores hepatic mass and function after injury or surgical resection through sequential priming, proliferative, and termination phases. Pharmacologic and metabolic interventions, including branched-chain amino acids, modulation of portal flow, and inhibition of mitogen-activated protein kinase kinase-4 (MKK4), may enhance regenerative signaling within this framework.

At the molecular level, liver regeneration proceeds through temporally overlapping phases involving priming, proliferation, and termination. The priming phase is characterized by the activation of quiescent hepatocytes through inflammatory signaling pathways. Cytokines such as tumor necrosis factor-α (TNF-α) and interleukin-6 (IL-6) activate transcriptional programs mediated by nuclear factor-κB (NF-κB) and signal transducer and activator of transcription-3 (STAT3), thereby enabling hepatocytes to reenter the cell cycle [14,15]. This priming stage is followed by the proliferative phase, in which hepatocyte replication is driven by mitogenic signals including hepatocyte growth factor (HGF)/c-Met and epidermal growth factor (EGF)/epidermal growth factor receptor (EGFR) pathways, as well as the Wingless (Wnt)/β-catenin and Hippo/Yes-associated protein (YAP) signaling cascades [16,17]. Finally, regeneration is halted by inhibitory mechanisms, including transforming growth factor-β (TGF-β) signaling and extracellular matrix remodeling, which collectively restore hepatic homeostasis and prevent uncontrolled proliferation (Figure 1) [18].

Portal hemodynamics represent a central upstream driver of regenerative signaling. Surgical techniques such as PVE take advantage of this physiologic principle by redirecting portal blood flow to the future liver remnant (FLR), thereby stimulating hypertrophy of the preserved parenchyma. PVE has become a widely adopted strategy for increasing the safety of extended hepatectomy in patients with insufficient residual liver volume [19]. However, volumetric increases do not necessarily correspond to functional improvement, highlighting the need to evaluate regeneration not only in terms of volume but also in terms of functional hepatic capacity [19].

### 3.1. Volumetric vs. Functional Liver Regeneration

Distinguishing between volumetric and functional liver regeneration is critical for preventing post-hepatectomy liver failure (PHLF), as traditional CT/MRI-based volumetry often overestimates metabolic reserve. Functional assessment tools, including hepatobiliary scintigraphy (HBS), indocyanine green (ICG) clearance, LiMAx testing, and functional MRI, provide necessary insight into metabolic, synthetic, and clearance capacity, which lags structural regeneration. Table 1 summarizes the characteristics, cut-off values, strengths, and limitations of each of these techniques based on published studies [20,21,22,23].

### 3.2. Associating Liver Partition and Portal Vein Ligation for Staged Hepatectomy

In this context, ALPPS deserves specific mention as a useful model to explore the physiology of liver regeneration in clinical practice. ALPPS is an advanced two-stage surgical technique designed to induce rapid hypertrophy of the future liver remnant (FLR), enabling the resection of otherwise unresectable hepatic tumors [24]. Here we discuss the regenerative physiology and clinical implications of ALPPS.

ALPPS accelerates future liver remnant hypertrophy by combining portal-flow redistribution with parenchymal transection, thereby integrating hemodynamic, inflammatory, and molecular regenerative signals. Mechanistic evidence from Zhang et al. supports the role of portal inflow redistribution and circulating proliferative factors [25], whereas Masuo et al. describe endothelial nitric oxide synthase activation as a contributor to accelerated regeneration [26]. The procedure redirects portal inflow toward the FLR, increasing shear stress and endothelial activation, while transection appears to amplify local inflammatory and wound-healing responses that include cytokine and growth-factor signaling. These mechanisms help explain why ALPPS can produce rapid FLR expansion, with studies reporting substantial hypertrophy within approximately 1–2 weeks [25,26,27].

However, the regenerative response is not purely volumetric. Studies emphasize that rapid FLR enlargement may outpace restoration of metabolic capacity, creating a clinically relevant dissociation between anatomical growth and functional recovery [28,29]. Experimental evidence further supports the concept that ALPPS induces distinct regenerative patterns compared with selective portal vein ligation [30]. Transcriptomic and functional data therefore suggest that ALPPS temporarily redistributes proliferative and metabolic programs between liver lobes, with the expanding FLR prioritizing cell-cycle activation, while the deportalized lobe may contribute transient metabolic support [28,29,30].

This distinction is clinically important because ALPPS can expand resectability and improve completion of staged hepatectomy, but its use remains constrained by morbidity, post-hepatectomy liver failure risk, and the need for careful patient selection. Kang and Schadde link hypertrophy and liver function to morbidity and mortality [29], whereas Chan et al. highlight the need to determine whether apparent FLR increase is truly functional [28]. Systematic-review evidence supports the broader clinical interpretation that ALPPS can improve completion of staged resection, while requiring careful assessment of risk [29]. Accordingly, contemporary evaluation of ALPPS should pair volumetric assessment with functional testing and should interpret technical refinements in the context of institutional expertise and tumor biology.

In summary, ALPPS offers a powerful strategy for increasing resectability in carefully selected patients, but its clinical use is constrained by the need to align rapid anatomical hypertrophy with adequate functional reserve. The most clinically relevant decisions therefore concern patient selection, objective assessment of FLR function, timing of second-stage surgery, and whether technical modifications can reduce morbidity without compromising oncological efficacy.

### 3.3. Role of Non-Parenchymal Cells

In addition to hepatocytes, non-parenchymal cells (NPCs) orchestrate liver regeneration by integrating inflammatory cues, growth-factor signaling, vascular adaptation, and extracellular matrix (ECM) remodeling [31,32,33]. Although hepatocytes provide the main proliferative output, regeneration depends on sustained crosstalk with Kupffer cells (KCs), liver sinusoidal endothelial cells (LSECs) and hepatic stellate cells (HSCs), which together initiate, pattern, and terminate the regenerative response.

#### 3.3.1. Individual Contributions of Non-Parenchymal Cells

KCs act as early sinusoidal sentinels after tissue loss. In response to altered portal flow, complement activation, and gut-derived signals, they release TNF-α and IL-6. These cytokines primarily prime quiescent hepatocytes to enter the G_1_ phase and become responsive to mitogenic ligands, including HGF and epidermal growth factor-family signals [31,32,33].

LSECs translate hemodynamic and biochemical changes into regenerative signals. Increased portal flow exposes them to shear stress, promoting nitric oxide production and sinusoidal perfusion [34]. LSECs also provide angiocrine cues, including VEGF-, HGF- and Wnt-linked pathways, that support hepatocyte proliferation and vascular expansion [34,35].

Hepatic stellate cells (HSCs) couple growth-factor availability to matrix remodeling. Beyond vitamin A storage and matrix maintenance, transiently activated HSCs regulate mitogen presentation during regeneration [33,36]. Through matrix metalloproteinases, ECM deposition and TGF-β signaling, they help remodel the scaffold during growth and restrain proliferation as tissue homeostasis is restored [32,36].

#### 3.3.2. Temporal Dynamics of Non-Parenchymal Cell Interactions

Regeneration unfolds as overlapping priming, proliferative, and termination programs [31,32]. During priming, inflammatory and flow-derived cues activate KCs and LSECs in parallel: KC-derived TNF-α and IL-6 confer hepatocyte competence, whereas LSEC-derived signals preserve perfusion and condition the regenerative niche [33,34].

During outgrowth, hepatocytes and NPCs reinforce one another. LSECs and HSCs provide growth factors, remodel ECM, and support angiogenesis, ensuring that hepatocyte expansion remains coupled to sinusoidal architecture and perfusion [33,35,36].

Termination is likewise niche-dependent. As liver mass is restored, antiproliferative cues, including TGF-β expression increases and the return of physiological NPC functions help return the microenvironment to quiescence [32,36]. NPCs are therefore best viewed as dynamic regulators of the onset, spatial organization, and cessation of regeneration [33].

### 3.4. Molecular Pharmacology

Recent advances in molecular pharmacology have expanded the therapeutic landscape by identifying intracellular signaling pathways that directly regulate hepatocyte proliferation. One of the most promising targets is mitogen-activated protein kinase kinase-4 (MKK4), a key component of stress-activated kinase signaling networks [37]. Experimental studies have demonstrated that inhibition of MKK4 releases a regenerative checkpoint in hepatocytes, thereby enhancing their proliferative capacity following liver injury or resection [37]. Structure-based drug discovery efforts led to the development of HRX215, a first-in-class small-molecule inhibitor of MKK4 [37].

Preclinical studies have shown that HRX215 significantly increases liver regeneration after hepatectomy in both murine and porcine models [37]. In mice, pharmacologic MKK4 inhibition significantly increased hepatocyte proliferation following partial hepatectomy without inducing uncontrolled cell growth in the intact liver [37]. In pigs, HRX215 accelerated the restoration of liver volume after major resection and, most strikingly, enabled survival after an otherwise lethal 85% hepatectomy [37]. This effect was associated with rapid expansion of functional liver mass and prevention of post-hepatectomy liver failure [37].

Importantly, the safety and pharmacokinetic profile of HRX215 have also been evaluated in humans. A first-in-human phase I clinical trial involving healthy volunteers demonstrated excellent tolerability and predictable pharmacokinetics without serious adverse effects [37]. These findings support the translational potential of MKK4 inhibition as a therapeutic strategy to prevent liver failure following major hepatectomy or small-for-size liver transplantation [37]. Beyond its pro-regenerative effects, MKK4 inhibition has also been shown to exert antisteatotic and antifibrotic effects in experimental models of chronic liver disease [37]. These additional properties may be particularly relevant in clinical contexts where hepatic steatosis or fibrosis impairs regenerative capacity, such as in patients with MASLD or chemotherapy-associated liver injury [37]. Research by Zwirner et al. stands out as one of the most innovative and promising, describing HRX215 as a first-in-class small-molecule inhibitor of mitogen-activated protein kinase kinase 4 (MKK4), a stress-activated upstream regulator within the c-Jun N-terminal kinase signaling axis [37]. The therapeutic rationale is biologically compelling: in the injured or partially resected liver, MKK4 appears to constrain hepatocyte cell-cycle progression. Pharmacologic inhibition of MKK4 may release a regenerative checkpoint and favor proliferation of the remnant parenchyma. The study further suggests that this effect is not simply mitogenic, but context dependent, with regenerative enhancement observed principally in damaged or resected livers rather than in the intact organ [37]. This distinction is important, as it supports the concept that HRX215 may amplify endogenous repair programs rather than induce indiscriminate hepatocyte proliferation. The reported antifibrotic and antisteatotic effects are also noteworthy, as they raise the possibility that MKK4 inhibition modifies the hepatic microenvironment in ways that could secondarily facilitate regeneration. The study demonstrates considerable strengths. It adopts a rigorous translational design spanning rational compound development, target engagement, mechanistic preclinical validation, efficacy testing in both murine and porcine hepatectomy models, and early human phase I evaluation. Particularly persuasive is the demonstration of survival benefit after extended hepatectomy in a large-animal model, which strengthens the biological and clinical plausibility of the approach beyond conventional rodent data alone. However, the limitations are equally substantial and should temper interpretation. Most importantly, the clinical component remains confined to phase I testing in healthy volunteers. Therefore, the study provides safety and pharmacokinetic information but no evidence of therapeutic efficacy in patients with impaired regenerative reserve. In addition, the principal efficacy signals derive from preclinical and surrogate endpoints, not from hard clinical outcomes such as post-hepatectomy liver failure, morbidity, or survival in humans. The duration of follow-up is also limited, leaving unresolved whether sustained MKK4 inhibition could have unintended consequences for inflammatory signaling, maladaptive repair, or oncologic risk. Accordingly, while HRX215 is an innovative and promising candidate, its clinical relevance remains provisional pending well-powered clinical trials in patients.

Taken together, current evidence indicates that liver regeneration is controlled by a complex network of hemodynamic, inflammatory, metabolic, and molecular signals. Interventions that enhance portal flow, modulate cytokine signaling, optimize metabolic conditions, or directly activate regenerative pathways can significantly impact the regenerative response. Clinical strategies, ranging from portal vein embolization and stem-cell therapy to nutritional supplementation and targeted molecular inhibitors, demonstrate the variety of approaches currently being investigated.

Despite these advances, translation of experimental insights into clinical practice remains incomplete. Many interventions that demonstrate strong regenerative effects in preclinical models still require validation in large, controlled clinical trials. Moreover, the heterogeneity of patient populations, underlying liver diseases, and surgical procedures complicate the interpretation of clinical outcomes.

## 4. Clinical Studies

Liver regeneration is a crucial factor in determining outcomes following hepatectomy, living donor liver procurement, and acute or chronic liver injury. The ability of the remaining liver tissue to regenerate and regain function directly impacts postoperative recovery and the risk of liver failure [38]. In recent years, there has been an increasing focus on strategies to enhance this regenerative response. These strategies range from technical adjustments in portal flow and perioperative care to pharmacological, nutritional, and molecular interventions. The studies summarized in Table 2 [19,39,40,41,42,43,44,45,46,47,48,49,50,51,52,53] demonstrate the growing interest in finding methods that not only promote rapid regrowth of liver volume but also improve functional recovery and enhance the safety of major liver resections, especially in high-risk settings. However, the existing evidence is varied, highlighting the importance of distinguishing between interventions with genuine therapeutic potential and those that primarily investigate predictive or mechanistic aspects of regeneration.

Interventions associated with enhanced liver regenerative capacity included portal vein embolization combined with autologous CD133^+^ bone marrow stem cells [40,44], branched-chain amino acid supplementation following PVE [19], pentoxifylline in patients with small future liver remnants [42], *N*-butyl cyanoacrylate (NBCA) plus iodized oil for pre-hepatectomy portal vein embolization [50], postoperative ursodeoxycholic acid administration in living liver donors [51], preoperative lifestyle optimization [52], and the TTK traditional Chinese medicine regimen [45]. Collectively, these interventions were associated with greater hypertrophy of the future liver remnant, accelerated volumetric expansion, improved postoperative liver function, or biologic signatures consistent with augmented regenerative activity [19,40,42,44,45,50,51,52].

CD133-positive stem/progenitor-cell therapy in chronic liver disease is best understood not as a strategy for durable hepatocyte replacement, but as a transient, trophic intervention that remodels the injured hepatic niche. Across clinical studies, infused autologous CD133^+^ or bone-marrow-derived cell products have shown acceptable short-term safety, but only modest or transient signals of efficacy, consistent with the view that their biological activity depends primarily on paracrine and immunomodulatory mechanisms rather than stable engraftment [54,55,56]. Mechanistically, transplanted cells are thought to act as short-lived “bioreactors” that deliver soluble mediators and extracellular vesicles to damaged tissue [57,58,59]. These secreted products can support endothelial repair and neovascularization through angiogenic pathways, including VEGF signaling, and can enhance hepatocyte survival, proliferation, and metabolic recovery through trophic cues such as HGF. Rather than repopulating the parenchyma, the cells therefore amplify endogenous repair programs.

A second, and probably central, component of this response is microenvironmental reprogramming. Stem-cell-derived factors and vesicular cargo can dampen inflammatory signaling, favor reparative macrophage polarization, restrain activation of hepatic stellate cells, and promote ECM turnover [58,59]. In this model, improvement in liver function reflects coordinated suppression of necroinflammation and fibrosis, together with stimulation of residual regenerative capacity.

Overall, the available evidence supports a niche-modifying paradigm for CD133^+^ cell therapy: the therapeutic signal is mediated by secreted cytokines, growth factors, and extracellular vesicles that recalibrate vascular, immune, and fibrogenic compartments, whereas direct conversion into functional hepatocytes appears limited [54,55,56,57,58,59]. This interpretation also explains why clinical responses, when observed, tend to be early and variable, and why future development is likely to depend on defining potency markers, optimizing cell dose and route, and comparing cell-based products with cell-free extracellular-vesicle-based approaches.

In parallel, observational studies have identified platelet serotonin and von Willebrand factor as potentially relevant modulators or predictors of regenerative performance. However, these findings do not constitute direct evidence of therapeutic efficacy [46,47].

By contrast, other investigations have failed to demonstrate a clear pro-regenerative effect or have only yielded inconclusive evidence. In fulminant hepatic failure, insulin plus glucagon did not improve survival, hepatic synthetic function, or histologic indices of regeneration [39]. Similarly, the comparison between isoflurane and propofol in donor hepatectomy did not reveal a significant difference in regenerated liver volume [53]. Synbiotic supplementation also produced equivocal results, with a possible benefit limited to a subgroup of patients without postoperative complications and no consistent overall advantage in the pilot trial [43]. Moreover, several studies, including postoperative volumetric analyses after hepatectomy and prognostic models based on remnant liver volume or the TACIA score (a prognostic system used to gauge disease severity and estimate short-term survival among subjects with acute-on-chronic liver failure) [49], primarily characterized determinants or predictors of regeneration rather than evaluating interventions designed to actively promote it [41,48,49].

Taken together, these studies indicate that liver regeneration may be influenced by a wide spectrum of strategies, including technical interventions, perioperative optimization, pharmacologic treatments, nutritional support, and emerging molecular approaches. Nevertheless, the available evidence remains markedly heterogeneous with respect to patient populations, underlying hepatic pathology, study design, and outcome measures. Many of the studies reporting favorable effects are limited by small sample sizes, single-center enrollment, reliance on surrogate volumetric endpoints, or highly selected cohorts, all of which constrain the external validity of their findings. In addition, a substantial proportion of the literature focuses on biomarkers or prognostic correlation rather than on interventions capable of improving clinically meaningful outcomes. Future investigations should therefore prioritize adequately powered multicenter randomized trials employing standardized definitions of regeneration, integrated volumetric and functional endpoints, and extended follow-up, to determine whether accelerated regenerative responses translate into reduced rates of post-hepatectomy liver failure, improved operative safety, and superior survival outcomes. Further research is also warranted to define the setting-specific efficacy of these strategies in steatotic or cirrhotic livers, living donor transplantation, and extended hepatectomy, as well as to explore biomarker-informed approaches for personalized regenerative support.

## 5. Conclusions: Impact in the Context of Translational Approaches and Future Studies

Liver regeneration is a remarkable example of physiological tissue repair in humans and is crucial for recovery after major hepatic injury or surgical resection. The studies discussed in this article show that hepatic regeneration is a complex, highly regulated process that can be influenced by multiple therapeutic strategies. Interventions targeting portal hemodynamics, metabolic support, inflammatory signaling, cellular therapy, and emerging molecular pathways demonstrate that regenerative capacity can be modulated in meaningful ways. One of the most clinically relevant strategies is optimizing the future liver remnant (FLR) before major resection. PVE remains the cornerstone of this approach, as it redirects portal blood flow toward the non-embolized liver segments and stimulates compensatory hypertrophy of the remnant parenchyma. However, the evidence discussed in this review indicates that technical interventions alone may not always be sufficient to ensure adequate regenerative recovery, particularly in patients with compromised liver quality due to steatosis, fibrosis, chemotherapy-associated injury, or metabolic comorbidities. Consequently, increasing attention has shifted toward combined strategies that integrate hemodynamic modulation with pharmacologic or metabolic support.

The addition of autologous CD133^+^ bone marrow-derived stem cells to PVE exemplifies a translational approach aimed at enhancing the endogenous regenerative response. Clinical studies have demonstrated that this combination can significantly increase the hypertrophy of the FLR and accelerate its growth rate compared with PVE alone, potentially allowing earlier and safer resection in patients with critically low residual liver volumes. These findings support the concept that circulating progenitor cells may participate in regeneration either through direct differentiation into hepatocyte-like cells or through paracrine mechanisms that modify the hepatic microenvironment. Although still limited by small patient cohorts and single-center experiences, this strategy highlights the broader therapeutic potential of regenerative medicine in hepatobiliary surgery.

Metabolic and nutritional interventions also represent a promising avenue for translational research. Branched-chain amino acid supplementation, for example, has been shown to improve functional regeneration and hepatic metabolic performance following PVE and major hepatectomy, suggesting that targeted nutritional support may facilitate recovery of liver function even when volumetric growth is modest. Similarly, postoperative administration of ursodeoxycholic acid has been associated with improved liver function parameters in living donors after hepatectomy, indicating that hepatoprotective agents may play a supportive role during the early regenerative phase. Lifestyle optimization before surgery has also emerged as an effective strategy, demonstrating that preoperative interventions aimed at reducing steatosis and improving metabolic fitness can enhance postoperative regeneration and graft outcomes. Beyond metabolic interventions, pharmacologic modulation of inflammatory signaling pathways has shown encouraging results. Pentoxifylline, a phosphodiesterase inhibitor with anti-inflammatory properties, has been shown to enhance regeneration in patients with small liver remnants, potentially through modulation of interleukin-6 signaling pathways. While the benefits appear most pronounced in patients with critically reduced residual liver volumes, these findings underscore the importance of cytokine-mediated signaling networks in the regulation of hepatocyte proliferation.

Recent translational advances have also identified novel molecular targets capable of directly modulating hepatocyte proliferation. Among these, inhibition of MKK4 represents one of the most innovative developments. Preclinical studies have demonstrated that pharmacologic inhibition of MKK4 with the small-molecule compound HRX215 markedly enhances hepatocyte proliferation and accelerates liver regeneration after partial hepatectomy. In large-animal models, this approach has even enabled survival following otherwise lethal extended hepatectomy, highlighting its potential to prevent post-hepatectomy liver failure. Importantly, early-phase clinical evaluation has shown favorable pharmacokinetics and safety profiles in healthy volunteers, providing the first step toward clinical translation. Nevertheless, the therapeutic efficacy of MKK4 inhibition in patients with impaired regenerative capacity remains to be established in well-designed clinical trials.

In parallel with therapeutic interventions, a growing body of research is identifying biomarkers capable of predicting regenerative performance and guiding clinical decision-making. Observational studies have shown that platelet-derived serotonin levels correlate with regenerative capacity and postoperative outcomes, suggesting that platelet signaling pathways play a significant role in hepatic repair. Similarly, perioperative dynamics of von Willebrand factor have been associated with platelet accumulation within the regenerating liver and with postoperative regenerative success, providing further evidence of the interplay between vascular biology, platelet activation, and hepatocyte proliferation. Although these markers currently serve primarily as predictors rather than therapeutic targets, their integration into perioperative risk stratification may enable more personalized approaches to liver surgery.

An additional area likely to influence future research in liver regeneration is the rapid development of high-resolution molecular and computational technologies. Single-cell and spatial transcriptomics are increasingly enabling the characterization of cellular heterogeneity within the regenerating liver and the spatial organization of signaling networks that regulate hepatocyte proliferation and tissue remodeling. When combined with multi-omics integration, including proteomic and metabolomic profiling, these approaches may provide a more comprehensive systems-level understanding of regenerative biology. In parallel, artificial intelligence-based analytical frameworks are being developed to integrate clinical, imaging, and molecular datasets to predict regenerative capacity and the risk of post-hepatectomy liver failure. Such technologies may ultimately facilitate more precise patient stratification and support the development of personalized perioperative strategies aimed at optimizing hepatic regeneration.

Despite these promising advances, several limitations must be acknowledged. The clinical studies available to date remain heterogeneous in terms of patient populations, underlying liver disease, surgical procedures, and outcome measures. Many trials rely on surrogate endpoints such as volumetric liver growth rather than clinically meaningful outcomes such as postoperative liver failure or long-term survival. Moreover, the small sample sizes and single-center designs of many studies limit the generalizability of their findings. For these reasons, translating experimental insights into routine clinical practice requires careful validation through adequately powered multicenter randomized trials. Moreover, a key challenge in therapeutic liver regeneration is to promote repair without facilitating tumor growth or recurrence. Because regenerative and tumor-associated programs can overlap, pro-regenerative interventions should be assessed for both efficacy and the risk of supporting residual malignant cells or recurrence [60]. This concern applies to HGF/c-Met and Wnt/β-catenin signaling, which support regeneration but are also linked to HCC progression, immune evasion, and treatment resistance. Excessive activation could therefore favor occult or residual tumor foci in susceptible contexts [61].

Aberrant Wnt/β-catenin activation, including recurrent CTNNB1 mutations, may provide proliferative advantages in HCC and should therefore be approached cautiously as a regenerative target [62].

Cell-based approaches also warrant caution, as trophic and immunomodulatory effects that support repair may remodel the tumor microenvironment or reduce surveillance of residual cancer cells. Rare concerns about ectopic or abnormal growth remain relevant for highly plastic transplanted cells [63].

MKK4 inhibition may enhance regeneration by releasing a restraint on hepatocyte proliferation [37,64]. However, because MKK4-related pathways intersect with stress responses, apoptosis, and tumor suppression, inhibition could allow damaged or premalignant hepatocytes to persist and expand [37]. 

Overall, pro-regenerative therapies should restore hepatic mass while limiting conditions that could favor tumor recurrence. Their oncological safety will likely depend on clinical context, residual disease burden, and engagement of mitogenic, immune, and checkpoint pathways.

Future research should therefore focus on integrating multiple complementary approaches to optimize liver regeneration, while ensuring the oncological safety of these approaches. Rational approaches include combining technical strategies such as portal flow modulation with pharmacologic agents, metabolic interventions, and regenerative therapies targeting specific molecular pathways. In addition, advances in systems biology, metabolomics, and biomarker discovery may allow the development of predictive models capable of identifying patients at high risk for postoperative liver insufficiency. Such approaches would enable personalized perioperative strategies tailored to the regenerative capacity of each patient.

**Figure 2:**
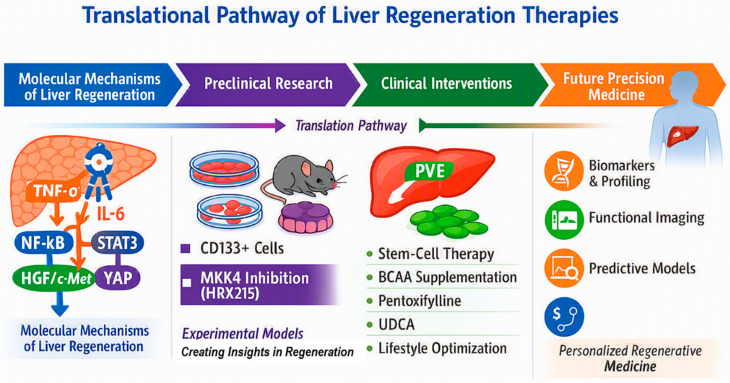
**Translational framework for therapeutic enhancement of liver regeneration**. Liver regeneration research increasingly follows a translational continuum that connects fundamental biological discoveries with clinical strategies aimed at improving outcomes after liver injury or resection. At the mechanistic level, regeneration is governed by complex networks involving inflammatory cytokines, growth-factor signaling, metabolic pathways, and platelet-mediated interactions that regulate hepatocyte proliferation and tissue remodeling. Insights from these pathways have enabled the identification of experimental therapeutic targets, including pharmacologic modulation of intracellular signaling pathways and regenerative cell-based approaches designed to amplify endogenous repair mechanisms. These discoveries have progressively informed clinical interventions such as portal vein embolization, stem-cell-assisted regenerative strategies, metabolic and nutritional support including branched-chain amino acid supplementation, pharmacologic modulation of inflammatory pathways, and perioperative optimization measures. Emerging molecular therapies, including inhibition of mitogen-activated protein kinase kinase-4 (MKK4), represent a new generation of targeted approaches aimed at directly stimulating hepatocyte proliferation in the context of liver injury or surgical resection. Future progress is expected to rely on the integration of molecular biomarkers, functional imaging, and individualized perioperative strategies to enable personalized regenerative support and expand the safety of major hepatobiliary surgery.

In conclusion, the expanding body of clinical and experimental evidence reviewed here supports the concept that liver regeneration is a dynamic and therapeutically modifiable process (Figure 2). Interventions ranging from portal vein modulation and stem cell therapy to nutritional supplementation and targeted molecular inhibitors have demonstrated the potential to enhance hepatic regenerative responses. Among these strategies, emerging targeted therapies such as MKK4 inhibition represent particularly promising candidates for future translational research. However, the integration of these approaches into clinical practice will require robust evidence demonstrating not only improved regenerative kinetics but also meaningful reductions in postoperative liver failure and improvements in patient survival. Ultimately, advances in molecular physiology and clinical pharmacology of liver regeneration may expand the boundaries of hepatobiliary surgery and improve outcomes for patients undergoing major hepatic resection or suffering from acute and chronic liver disease.

## Figures and Tables

**Table 1:** Characteristics, cut-off values, strengths, and limitations of volumetric and functional liver regeneration.

Modality	Key Parameters Assayed	Clinical Cut-Off Values for Safe Resection/PHLF Risk	Major Advantages	Key Limitations	Reference
HBS	Technetium-99m mebrofenin hepatobiliary scintigraphy uptake	FLR function ≤ 2.69–2.72%/min/m^2^	Segment-specific, unaffected by jaundice	Ionizing radiation, lower availability	[20]
ICG	Retention at 15 min (ICG-R_15_)	ICG-R_15_ > 15% (general) or >8.5% (cirrhotic)	Bedside test, cost-effective	Global metric, high bilirubin affects results	[21]
LiMAx	^13^C-methacetin metabolism (CYP1A2)	Preoperative LiMAx < 140 μg/kg/h	Real-time, independent of biliary disorders	Global metric, specialized hardware	[22]
fMRI	Gd-EOB-DTPA enhancement	Relative enhancement <47.7%	Combines tumor staging with regional function	High cost, scan variability	[23]

Abbreviations: CYP1A2, cytochrome P450 1A2; FLR, future liver remnant; fMRI, functional magnetic resonance imaging; Gd-EOB-DTPA, gadolinium ethoxybenzyl diethylenetriamine pentaacetic acid; ICG-R_15_, indocyanine green retention rate at 15 min; LiMAx, Liver Maximum Capacity; PHLF, post-hepatectomy liver failure.

**Table 2:** Summary of main studies on liver regeneration ordered chronologically.

Author/Year/Ref	Method	Findings	Conclusions	Limitations
Harrison et al., 1990 [39]	RCT in 18 patients with fulminant hepatic failure; insulin plus glucagon infusion versus control.	No reduction in mortality and no improvement in hepatic synthetic function or histologic regeneration.	Insulin and glucagon did not stimulate liver regeneration in this setting.	Very small cohort of 18 patients, enrolled at a late stage of illness; heterogeneous study population; single-center design.
Fürst et al., 2007 [40]	Prospective comparative study of PVE with or without autologous CD133^+^ stem cells in patients with low FLRV.	Greater absolute and relative FLRV increase and faster daily liver growth in the stem-cell group.	Adding CD133^+^ bone marrow stem cells to PVE enhanced liver regeneration compared with PVE alone.	Very small, non-randomized cohort of 13 patients; baseline imbalances between study and control groups; short follow-up focused on volumetric gain rather than long-term outcomes; and no data on the precise biological mechanism of the cellular treatment.
Zappa et al., 2009 [41]	Preoperative and postoperative day-7 CT volumetric analysis after right hepatectomy in 27 patients.	A 64% increase in remnant liver volume; lower segmental regeneration when the middle hepatic vein was harvested.	Early liver regeneration varies by segment and is influenced by venous outflow.	Short follow-up and focus on volumetric rather than functional changes; retrospective data collection; manual image segmentation; and variable intervals between preoperative imaging and surgery.
Petrowsky et al., 2010 [42]	Double-blind RCT in 101 patients undergoing major liver resection; pentoxifylline versus placebo.	Better volumetric regeneration in small remnant livers, lower AST levels, but more drug-related adverse events.	Pentoxifylline may enhance regeneration in small liver remnants, likely through IL-6 signaling.	Single-center investigation in a non-cirrhotic cohort; sample size powered for radiological volume rather than clinical outcomes; reliance on volumetry rather than functional assessment; and exclusion of high-risk cases.
Rayes et al., 2012 [43]	Double-blind randomized pilot study in 19 patients; synbiotics versus placebo fiber after right hepatectomy.	Overall liver function was similar between groups; a possible benefit appeared in patients without complications, but numbers were small.	Synbiotics may improve postoperative liver function, but larger studies are needed.	Small, 19-patient, single-center pilot study; complex multi-strain intervention; heterogeneous underlying pathologies; and preliminary, feasibility-focused data.
Knoefel et al., 2013 [44]	Study of PVE combined with CD133^+^ bone marrow stem cells versus PVE alone in patients with critically low FLRV.	Significantly greater FLRV increase, faster daily growth, and shorter time to surgery with stem-cell support.	PVE plus stem-cell therapy may make resection safer in high-risk patients.	Low efficiency of stem-cell homing; potential oncogenic risks; and limited data on long-term outcomes.
Li et al., 2014 [45]	RCT in 144 patients with chronic hepatitis B-associated liver failure; traditional Chinese medicine regimens versus control.	Lower mortality and improvement in selected biochemical parameters in the treated groups, especially the TTK group.	The TTK intervention may improve survival and support liver function and regeneration.	Limited generalizability due to a Chinese-only cohort; difficulty isolating the mechanism of the multi-herb formula; small sample size; reliance on surrogate markers; and short 8-week follow-up.
Starlinger et al., 2014 [46]	Perioperative observational clinical study in 60 patients undergoing liver resection; platelet serotonin monitoring.	Low preoperative intraplatelet serotonin was associated with delayed regeneration and higher risk of liver dysfunction.	Platelet serotonin may serve as a predictive marker of liver regeneration and postoperative outcome.	Small, heterogeneous sample (n = 60); differences between cohorts in preoperative chemotherapy exposure and cirrhosis; serotonin estimates based on mathematical calculations rather than direct measurement; and correlative rather than causal findings.
Beppu et al., 2015 [19]	RCT in patients undergoing PVE followed by hepatectomy; BCAA supplementation versus control.	Improved functional liver regeneration and liver function in the BCAA group.	BCAAs improve functional regeneration and liver function after PVE and major hepatectomy.	Small sample size and specific Japanese patient cohort; reliance on surrogate metrics for functional hypertrophy; focus on a single surgical procedure; and short follow-up for a nutritional intervention.
Starlinger et al., 2018 [47]	Clinical study of perioperative von Willebrand factor dynamics in patients undergoing liver resection.	An early vWF surge was associated with adequate regeneration; high preoperative levels predicted postoperative dysfunction.	vWF is involved in liver regeneration and may be useful for preoperative risk stratification.	Potential selection bias and confounding from systemic comorbidities that may independently elevate vWF. The ≥182% cutoff also requires validation across broader patient populations and may be confounded by tumor progression in oncologic cases.
Gong et al., 2019 [48]	Clinical study in 125 patients with hepatocellular carcinoma after hemihepatectomy; serial assessment of remnant liver volume.	%FLRV below 42.7% predicted liver failure; FLRV, %FLRV, and cirrhosis influenced regeneration.	Functional remnant liver volume is a strong predictor of regeneration and post-hepatectomy liver failure.	Small, single-center patient cohort; retrospective and observational study design.
Wang et al., 2020 [49]	Prognostic study in 308 patients with HBV-ACLF; development of the TACIA score using regeneration-related biomarkers.	Creatinine, age, bilirubin, AFP, and INR were independent predictors; the model showed good AUROC performance.	The TACIA score effectively predicts short-term survival in patients with HBV-ACLF.	Retrospective, single-center design focused solely on HBV etiology; short 90-day evaluation window; and volatility of biomarkers such as alpha-fetoprotein.
Luz et al., 2021 [50]	RCT of pre-hepatectomy portal vein embolization; NBCA plus iodized oil versus PVA plus coils.	Faster and greater liver regeneration with NBCA; more patients became eligible for surgery earlier.	NBCA plus iodized oil was superior to PVA plus coils in promoting future liver remnant hypertrophy.	Small sample size; single-center design; reliance on CT volumetry rather than functional assessment; limited heterogeneity in tumor types; and use of a fixed embolic ratio.
Aloun et al., 2023 [51]	Prospective double-blind RCT in 60 living liver donors; postoperative UDCA versus no UDCA.	Improved INR and several liver function test results in the UDCA group.	Postoperative UDCA improved liver tests in living donors after hepatectomy.	Small sample size (n = 60); single-center design; highly homogeneous donor cohort; short-term intervention; and reliance on surrogate laboratory markers rather than long-term, direct volumetric measurements of liver growth.
Gupta et al., 2023 [52]	RCT in living donors; preoperative lifestyle optimization versus usual routine.	Greater volumetric regeneration, less steatosis, less blood loss, and less early graft dysfunction in recipients.	Lifestyle optimization improved donor liver regeneration and recipient outcomes.	Small sample size (n = 62); single-center design; short two-week intervention; inclusion limited to healthy donors with minimal steatosis; and potential performance bias due to lack of blinding.
Abhinaya et al., 2025 [53]	Randomized controlled pilot trial in 60 donor hepatectomy cases; isoflurane versus propofol.	RgLV at postoperative day 14 was comparable between groups, with no significant differences.	Propofol and isoflurane did not appear to have different effects on liver regeneration after donor hepatectomy.	Small sample size (n = 60); single-center design; 14-day outcome window; and restriction to healthy living donors, limiting generalizability to high-risk patients.

Abbreviations: AFP, alpha-fetoprotein; AST, aspartate aminotransferase; AUROC, area under the receiver operating characteristic curve; BCAA, branched-chain amino acid; CT, computed tomography; FLRV, future liver remnant volume; HBV-ACLF, hepatitis B virus-associated acute-on-chronic liver failure; IL-6, interleukin-6; INR, international normalized ratio; NBCA, N-butyl cyanoacrylate; PVA, polyvinyl alcohol; PVE, portal vein embolization; RCT, randomized controlled trial; RgLV, regenerated liver volume; TTK, tonifying the kidney to promote liver regeneration and repair by affecting stem cells and their microenvironment; UDCA, ursodeoxycholic acid; vWF, von Willebrand factor.

## Data Availability

No new data were created or analyzed in this study. Data sharing is not applicable to this article.

## Abbreviations

The following abbreviations are used in this manuscript:

AFP	Alpha-fetoprotein
ALB	Albumin
ALP	Alkaline phosphatase
ALPPS	Associating liver partition and portal vein ligation for staged hepatectomy
ALT	Alanine aminotransferase
AST	Aspartate aminotransferase
AUROC	Area under the receiver operating characteristic curve
BCAA	Branched-chain amino acid
CCC	Cholangiocellular carcinoma
CT	Computed tomography
CTx	Chemotherapy
EGF	Epidermal growth factor
EGFR	Epidermal growth factor receptor
FLR	Future liver remnant
FLRV	Future liver remnant volume
HBV	Hepatitis B virus
HBV-ACLF	Hepatitis B virus-associated acute-on-chronic liver failure
HCC	Hepatocellular carcinoma
HGF	Hepatocyte growth factor
HRX215	Mitogen-activated protein kinase kinase-4 inhibitor (investigational compound)
HSC(s)	Hepatic stellate cell(s)
ICG	Indocyanine green
IL-6	Interleukin-6
INR	International normalized ratio
KC(s)	Kupffer cell(s)
LD	Liver dysfunction
LR	Liver regeneration
LSEC(s)	Liver sinusoidal endothelial cell(s)
MAPK	Mitogen-activated protein kinase
MKK4	Mitogen-activated protein kinase kinase-4
mCRC	Metastatic colorectal cancer
NBCA	N-butyl cyanoacrylate
NF-κB	Nuclear factor-κB
NPC(s)	Non-parenchymal cell(s)
PDR	Plasma disappearance rate
PH	Partial hepatectomy
POD	Postoperative day
PT	Prothrombin time
PVE	Portal vein embolization
PVA	Polyvinyl alcohol
RCT	Randomized controlled trial
RgLV	Regenerated liver volume
RLV	Remnant liver volume
STAT3	Signal transducer and activator of transcription-3
TBIL	Total bilirubin
TGF-β	Transforming growth factor-β
TNF-α	Tumor necrosis factor-α
TTK	Tonifying the kidney to promote liver regeneration and repair by affecting stem cells and their microenvironment
TQD	Tonifying qi and detoxification
UDCA	Ursodeoxycholic acid
vWF	von Willebrand factor
Wnt	Wingless signaling pathway
YAP	Yes-associated protein
